# Applications and Implications of Neutral *versus* Non-neutral Markers in Molecular Ecology

**DOI:** 10.3390/ijms12063966

**Published:** 2011-06-14

**Authors:** Heather Kirk, Joanna R. Freeland

**Affiliations:** Department of Biology, Trent University, Peterborough, Ontario K9J 7B8, Canada; E-Mail: heatherkirk@trentu.ca

**Keywords:** molecular markers, natural selection, genetic diversity, genetic drift, genetic differentiation, gene expression, next-generation sequencing

## Abstract

The field of molecular ecology has expanded enormously in the past two decades, largely because of the growing ease with which neutral molecular genetic data can be obtained from virtually any taxonomic group. However, there is also a growing awareness that neutral molecular data can provide only partial insight into parameters such as genetic diversity, local adaptation, evolutionary potential, effective population size, and taxonomic designations. Here we review some of the applications of neutral *versus* adaptive markers in molecular ecology, discuss some of the advantages that can be obtained by supplementing studies of molecular ecology with data from non-neutral molecular markers, and summarize new methods that are enabling researchers to generate data from genes that are under selection.

## 1. Introduction

The contributions that molecular biology has made to ecological research over the past two decades are phenomenal, and have created the relatively new field that is known as molecular ecology. During that time, methods for genetically characterizing individuals, populations, and species have become almost routine, and have provided us with fascinating new insights into the ecology and evolution of virtually all taxonomic groups [1]. Molecular markers allow us, among other things, to quantify genetic diversity [2,3], track the movements of individuals [4,5], measure inbreeding [6,7], identify species from mixed samples (for example soil samples or gut contents) [8,9], characterize new species [10,11] and retrace historical patterns of dispersal [12,13]. Building on these accomplishments, the field of molecular ecology continues to evolve, and among the more recent developments is a growing awareness that neutral molecular data—on which the majority of published studies in molecular ecology are based–can provide only partial insight into parameters such as genetic diversity, local adaptation, evolutionary potential, effective population size, and taxonomic designations [14–16] (but see [17,18]).

Biologists constantly strive to better understand evolution, and this quest is an important reason why we increasingly seek the information that can be obtained from adaptive genes (*i.e.*, genes that directly influence fitness). The relatively recent focus on non-neutral (adaptive) markers in molecular ecology can be further attributed to the potential practical applications of this approach, for example the identification of disease-causing genes or genes that can improve crop yields. In addition, there is growing concern over the rate at which environmental change is now occurring around the world. Species have three options that may allow them to survive rapidly changing environments: dispersal, phenotypic plasticity, or adaptation. If a species is unable to disperse from its native range to other suitable habitats, and is incapable of a plastic response, its survival will require rapid adaptive change which is possible only if an adequate level of adaptive genetic variation has been maintained [19,20]; therefore neutral and adaptive genetic diversity will likely have different impacts on long-term survival because only one (adaptive diversity) will allow a population to adapt to changing environmental conditions [21,22].

Another reason for the growing interest in adaptive variation is more practical: we are increasingly able to develop and utilize molecular markers that allow us to characterize non-neutral genomic regions. In recent years researchers have not only been able to identify those gene regions that are most likely to be under selection in natural populations, but in some cases have then been able to identify the function of adaptive genes and, ultimately, to link phenotype to genotype across a range of environmental conditions (Table 1). Recent advances in our technological capabilities to capture markers at hundreds or thousands of loci, combined with ongoing improvements in the abilities of statistical tools and software to tease apart expectations based on neutral *versus* non-neutral models of evolution, have led to an explosion in the number of studies that incorporate or target non-neutral markers for questions in the fields of population genetics, molecular ecology, and evolutionary biology. This relatively recent ability to identify DNA regions and even genes under the influence of selection is rapidly closing the gap between molecular biologists who study mechanisms of gene transcription, translation, and regulation, and those biologists who are interested in addressing the role of selection in shaping biodiversity.

## 2. Adaptive Genes and Genetic Diversity

Genetic diversity is a critical measure in population genetics because it can tell us a great deal about the current and likely future health of a population: low levels of genetic diversity can lead to inbreeding depression in the short-term, and to reduced evolutionary potential in the longer term. To date, the vast majority of genetic diversity estimates have been based on neutral markers. Although these data continue to provide us with invaluable insights into the overall levels of genetic variation within populations, in recent years they have been increasingly supplemented with data from adaptive genetic variation. Below, we shall discuss some of the ways in which these more recently acquired data have improved our understanding of inbreeding and evolutionary potential.

### 2.1. Inbreeding

Inbreeding occurs when individuals mate with their relatives. Depending on how closely related the parents are, the resulting inbred offspring will have a moderate to large proportion of alleles that are identical by descent, in other words they will exhibit a genome-wide increase in homozygosity relative to outbred individuals. This often leads to a reduction in fitness through a phenomenon that is known as inbreeding depression. Two processes can lead to inbreeding depression: dominance and overdominance. Dominance refers to the unmasking of deleterious recessive alleles that accompanies the overall increase in homozygosity; this occurs when unfavourable alleles that formerly occurred primarily in heterozygous individuals become more prevalent in a homozygous state, and therefore their deleterious effects are manifested. Overdominance, also known as heterozygote advantage, means that individuals that are heterozygous at a particular locus have higher fitness than individuals that are homozygous for either allele; the general increase in homozygosity that accompanies inbreeding means that beneficial heterozygotes become less common, once again reducing fitness.

Quantifying genome-wide heterozygosity is impractical, and is therefore typically inferred from a subset of loci such as microsatellites (e.g., [33]). Similarly, there are logistical constraints to quantifying fitness based on lifetime reproductive success, and therefore one or more surrogate measures such as clutch size, sperm count, or seed production is most commonly used (e.g., [34]). Multilocus genotype data and fitness estimates can be combined to test for heterozygosity fitness correlations (HFCs), which occur when there is a correlation between overall heterozygosity and a measure of fitness; a positive HFC suggests that low heterozygosity is reducing fitness within a population. Although a correlation between heterozygosity and fitness is widely accepted as evidence of inbreeding depression (reviewed in [35]), others have argued that HFCs that are based on only a small number of neutral markers may not reflect inbreeding depression because they are unlikely to represent genome-wide changes in homozygosity [36]. This was recently illustrated by a study of a free-ranging pedigreed population of the endangered takahe (*Porphyrio hochstetteri*) in which even relatively large numbers (>20) of microsatellite loci provided imprecise estimates of individual genome-wide heterozygosity [37]. The shortcomings of inbreeding estimates based on HFCs may therefore be twofold: first, it may be inappropriate to extrapolate genome-wide estimates of heterozygosity from small numbers of loci, and second, such extrapolation may be further weakened by the fact that heterozygosity is typically calculated on a subset of alleles that are neutral, and that have no functional significance in terms of adaptation and fitness (but see [38]).

An alternative approach to studying inbreeding depression is to seek specific information about its underlying molecular basis [39]. The first whole-genome study on the relationship between inbreeding and gene expression was done on *Drosophila melanogaster* [40]. The authors of that study compared gene expression in inbred and outbred lines of *D. melanogaster*, and determined that inbreeding changes transcription levels for a number of genes. The genes that showed differential expression in inbred lines were disproportionately involved in metabolism and stress responses, for example heat shock protein genes, which are involved in stress response, were upregulated more (*i.e.*, expressed in greater amounts) in inbred flies. This suggests that inbreeding acts like an environmental stressor that confers metabolic costs, and therefore leaves less energy for reproduction; in other words, inbreeding reduces fitness because stress responses are using energy that would otherwise be allocated to reproduction. This effect was even more pronounced when flies were placed in a high temperature environment, which conferred even greater stress and had the effect of further increasing the differential expression of heat-shock protein and metabolism genes in inbred *versus* outbred flies [41]. This latter study supports the idea that inbred organisms will be particularly challenged in stressful environments, and is consistent with an earlier study which found that inbreeding depression is on average 6.9 times higher for mammals in the wild compared to mammals that are kept in the relatively stress-free confines of captivity [42].

Demontis *et al.* [43] extended the study of gene expression in inbred *Drosophila* by investigating 40 SNPs in coding regions of genes that were identified in the earlier studies as being differentially expressed in inbred and outbred lines. They compared fast inbred lines, which took one generation to reach a predefined level of inbreeding, with slow inbred lines, which took 19 generations to reach the same level of inbreeding. Specifically, they wished to test the hypothesis that slow inbreeding leads to lower levels of inbreeding depression compared to fast inbreeding, because the former may allow more efficient purging of deleterious alleles and/or or more efficient selection for heterozygotes. They found a significantly higher level of genetic variation in the slow inbred lines compared to the fast inbred lines, including fewer homozygotes, and concluded that higher genetic diversity in slow inbred lines is a result of more efficient selection for heterozygotes (balancing selection) compared to the fast inbred lines. This indicates that, at least in this case, overdominance is likely the primary mechanism of inbreeding depression. Studies such as these strongly suggest that the use of “omic” approaches (e.g., genomics, proteomics) to unravel some of the cellular mechanisms behind inbreeding depression will feature more prominently in the near future [44].

### 2.2. Evolutionary Potential

Populations with low levels of genetic diversity should be less able to adapt to novel selection pressures, because a limited gene pool should decrease the likelihood that adaptive alleles will be present within a population. This expectation has been upheld by a growing number of studies. For example, populations of *Mercurialis annua* with reduced genetic diversity following range expansion had a reduced ability to respond to natural selection on a key life history trait [45]. In another example, laboratory populations of an estuarine crustacean (*Americamysis bahia*) with low genetic diversity had reduced fitness compared to populations with high genetic diversity; under stressful conditions the majority of low diversity populations went extinct, whereas populations with high genetic diversity were able to survive, albeit with reduced population sizes and less frequent reproduction [46].

The genetic diversity within populations is influenced by a range of factors, the most important of which is effective population size (*N*_e_), a measure that was introduced in the 1930s by Sewall Wright [47,48] who defined it as “the number of breeding individuals in an idealized population that would show the same amount of dispersion of allele frequencies under random genetic drift or the same amount of inbreeding as the population under consideration” [48]. In other words, the *N*_e_ of a population reflects the rate at which genetic diversity will be lost following genetic drift: only in an ideal population (sensu Wright) will the loss of genetic diversity as a result of drift occur at a rate that is commensurate with its actual population size. Understanding *N*_e_ is relevant to predictions about the viability of populations, because populations with low *N*_e_ are expected to have little evolutionary potential, and hence may be unable to respond to changing environmental conditions. However, this leaves us with a conundrum: estimates of *N*_e_ that are derived from molecular genetic data must be based on neutral markers (most commonly microsatellites) because *N*_e_ reflects the rate at which genetic drift—not selection—is altering allele frequencies from one generation to the next. As a result, *N*_e_ may tell us little about adaptive potential. This was recently illustrated by a study of the evolution of pesticide resistance in populations of the fruitfly *Drosophila melanogaster*, which concluded that resistance alleles have evolved quickly and repeatedly within multiple populations [49]. The authors of that study argue that such extensive evolutionary change would require a substantially larger (>100-fold) effective population size than had previously been identified. They further suggest that this discrepancy arises from the fact that estimates of *N_e_* are usually derived from levels of standing variation which in turn is influenced by long-term population dynamics, whereas short-term effective population sizes are more relevant for rapid adaptation, and these may be much closer to *N*_c_.

To date, most studies that have managed to quantify adaptively important genetic diversity have been based on three gene families whose diversity is maintained by balancing selection: major histocompatibility complex (MHC) loci in vertebrates [50], self-incompatibility loci in plants [51], and sex loci in Hymenoptera [52]. However, these families collectively represent only a modest proportion of all adaptive genetic variation. Furthermore, it is not entirely clear what impacts the loss of diversity at these loci may have on the survival of populations, because the interactions between selection and drift have often resulted in correlations between levels of MHC variation and variation at neutral loci [53]. An understanding of the link between neutral and adaptive diversity, and their collective influence on long-term survival, must therefore be based on a larger number of adaptive genes. There are a number of different methods that can be used to identify these genes, some of which are outlined in Box 1. Depending on the methods used, researchers may be able to identify genes that appear to be under selection (candidate genes) on the basis of allele frequency distributions. An example of this was reported in a study of threespine stickleback (*Gasterosteus aculeatus*), in which the authors used next generation sequencing to genotype 100 fish from each of three freshwater and two oceanic populations at 45,000 single nucleotide polymorphisms (SNPs) [54]. The population genetic signal from neutral markers indicated that a panmictic oceanic population gave rise to freshwater populations multiple independent times, while outlier loci provided evidence that balancing and divergent selection occurred in parallel genomic regions in different freshwater populations with independent origins. A number of candidate genes involved in differentiation were identified, providing the basis for further studies of adaptation at these loci.

Box 1 **Genomics techniques to generate sequence, genotype and gene expression data**

Recent and ongoing developments in both analytical and statistical tools have advanced the capabilities of molecular ecologist and evolutionary biologists to address complex questions regarding population genetic structure and processes of adaptation. We do not intend here to thoroughly review new methodologies that can be used to identify, characterize, and analyse genomic information, because comprehensive reviews have been published elsewhere (e.g., [64–66]). However, it is fitting to briefly summarize a few of the relatively recent techniques that are facilitating the large scale analysis of both neutral and non-neutral markers, including next generation sequencing (NGS), novel genotyping strategies, and strategies for studying gene expression.

NGS methods permit the rapid sequencing of genomic DNA, mRNA, or cDNA at relatively low (and rapidly falling) costs. Using various platforms (the best known of which include those manufactured by Illumina, Roche and ABI), it is possible to generate hundreds of thousands to millions of reads from a single lane, with read lengths between approximately 30 and several hundred base pairs. Each lane may contain a single sample, or it may contain pooled samples, each of which may be labeled with a unique nucleotide tag. This permits the development of large databases of genomic information from model- and non-model organisms alike, and is also driving the demand for the expansion of the bioinformatics field. Third generation sequencing technologies, set to be released over the next several years, promise to increase read length to approximately 10,000 bp at much greater speed (e.g., [67]), which will greatly increase the ease and accuracy of *de novo* assembly.

In many studies of non-model organisms, NGS is currently used for marker discovery because the comparison of whole genomes or whole transcriptomes (or expressed sequence tags; ESTs) remains expensive, time consuming, and analytically daunting. Recent advances in genotyping technologies have also improved the economy of including a large number of loci in various types of studies (reviewed by [68]). Both single nucleotide polymorphisms (SNPs) and microsatellites (or simple sequence repeats; SSRs) remain the most commonly used types of markers, particularly for population genomics studies, or gene association studies. SNPs are useful because they are ubiquitous in most genomes (and can therefore yield excellent coverage of the genome), and are relatively cost-effective and easy to genotype because most are biallelic (only two alternative nucleotides at a single SNP). A great variety of different commercial genotyping methods are offered; these include commercially available SNP microchips for model organisms and common agricultural species, and commercial genotyping services, such as the GoldenGate Assay offered by Illumina [69]. SNPs can also be genotyped at a small scale in-house using commercially available kits, but the costs of doing so are generally higher than outsourced options. On the other hand, microsatellites can yield a much greater amount of information per locus because they often exhibit a large number of alleles. However they are generally more time consuming and expensive than SNPs to genotype (with a smaller variety of commercial options), and this tends to be reflected in less extensive genome coverage in studies incorporating microatellites.

Although the genome coverage for microsatellites is lower than that of SNPs, they continue to be widely used because of their tremendous utility in molecular ecology [70]. One limitation of microsatellites, however, is the time and expense required for *de novo* development [71,72]; this is particularly problematic in some taxonomic groups such as Lepidoptera [73]. In addition, the PCR primers that are used to amplify microsatellite loci are often species-spec ific and therefore cannot be used on multiple taxa (Ellis and Burke, 2007). However, a more recently developed approach for characterizing microsatellites uses publicly available expressed sequence tag (EST) databases. An EST represents a single sequencing run starting from one end of a cDNA, and yields a sequence that is a small portion of the expressed gene. The growing use of NGS means that EST databases such as the National Center for Biotechnology Information (NCBI) EST database (dbEST; [74]) can be increasingly used for efficiently developing so-called EST-SSRs for a wide variety of taxa (reviewed in [75]). The evidence so far suggests that EST-SSRs are more likely to be transferrable between taxa than the more traditionally-developed SSRs which are isolated from a species’ genome in an anonymous manner [76–78]. EST-SSRs may also facilitate the generation of molecular markers that are directly associated with a trait of interest, and are therefore increasingly common in studies of molecular ecology (reviewed in [79]).

Advances also continue to be made in the area of gene expression studies, which can be helpful for the identification of important functional genes that may be under selection. NGS now permits direct transcriptome sequencing (RNA-seq), which can provide quantitative information on gene expression in different tissues, individuals, or populations. The number of reads generated for any particular transcript is expected to be proportional to the level of transcription, so that the so-called “read depth” can be used to generate information on relative transcription levels from different samples. Custom microarrays can also be commercially constructed for non-model organisms at reasonable costs; an investigator needs only to input the desired oligonucleotide probe sequences into an online database (these are often designed based on an initial NGS sequencing run of the transcriptome), and arrays are printed using an automated system. Once identified, the expression of individual candidate genes in various individuals and/or tissues can be verfied using quantitative PCR (qPCR).

The search for a link between adaptive genes and evolutionary potential has been complicated in recent years by the growing awareness that gene expression can play a role in the adaptive divergence of populations. Gene expression is influenced by both genetic and environmental factors, with the relevant genetic factors being changes in either regulatory genes or *cis*-regulatory regions (as opposed to protein-coding regions) of functional genes. Examples in the literature which show how gene expression can influence the adaptive divergence of populations are growing. In one study, at least 4% of the compared transcriptome significantly differed between two sympatric ecotypes of the marine snail *Littorina saxatilis.* One of the identified transcripts was cytochrome c oxidase subunit I (COI), a mitochondrial gene involved in energy metabolism. This gene was overexpressed in the lower shore ecotype which is subject to the strongest wave action, and which therefore may need a particularly effective energy supply [55]. In another study, this time on the model species *Drosophila melanogaster*, population differentiation of gene expression (measure as *Q*_ST_, or quantitative trait variation; see [56]) was not correlated with *G*_ST_ (an analogue of *F*_ST_ [57]) when based on all nucleotide polymorphisms; however, a correlation between *Q*_ST_ and *G*_ST_ was found when based on a more specific comparison in which *G*_ST_ was based solely on nucleotide differences in the 5′ coding regions of genes, in other words the regions that contain regulatory sequences [58].

Overall, neutral molecular markers have some clear advantages when used to estimate the genetic diversity of populations: they are relatively easy to characterize, and they can provide unbiased estimates of random processes such as genetic drift [59,60]. However, microsatellites, which are currently the most widely used markers for inferring genetic diversity [1], may not accurately reflect the genome-wide genetic diversity of natural populations [61,62], in part because a relatively small number of microsatellite loci are usually characterized. Although neutral markers will undoubtedly continue to play an important role in at least initial estimates of heterozygosity, we will likely see in the future a greater emphasis on whole genome scans, patterns of gene expression, and the functional analyses of genes [44,63]. (See also Box 1).

## 3. Genetic Differentiation

One of the most important determinants of microevolutionary change is gene flow between populations, because migrants typically increase *N*_e_ by introducing novel alleles, whereas isolated populations are more susceptible to the effects of genetic drift and therefore loss of alleles. Gene flow can therefore be considered an evolutionary facilitator because it increases the gene pool upon which selection can act. Conversely, gene flow can be viewed as an evolutionary deterrent because the continued introduction of alleles may counter local adaptation; the latter has been proposed as one explanation for the limits of species’ ranges ([80], and references therein). Thus, there exists the potential for tension between adaptation and gene flow, particularly at range margins, and the outcome will partly depend on the strength of selection pressure *versus* the extent of gene flow. This may result in different patterns of differentiation in adaptive *versus* non-adaptive genes, although before exploring that possibility, it is necessary to consider how we may determine which genes appear adaptive across a landscape.

### 3.1. Identifying Adaptively Divergent Genes

Migration and drift are expected to have approximately equal effects on all neutral loci, whereas the effects of selection will vary between neutral and non-neutral loci. All neutral loci may therefore show similar levels of genetic divergence among populations (once variable mutation rates have been accounted for), whereas non-neutral loci (or loci linked to non-neutral loci) are expected to show anomalous levels of divergence. These anomalous levels may be either unusually high or unusually low, depending on the type of selection that the relevant genes have been subjected to; for example, directional selection will increase population differentiation if different alleles are selected for in different populations, whereas balancing selection may decrease population differentiation by maintaining the same suite of alleles in multiple populations. A comparison of multiple measures of population differentiation, each based on a different locus, may reveal a marker with unusual levels of differentiation; this is often referred to as an outlier, and if the marker is found within a coding region, the latter may be considered a candidate gene [81,82]. An outlier may be used to identify a genetic region that is either directly under selection, or is linked to a gene that is under selection [83,84]. Approaches for using genome scans to identify markers of potential adaptive significance are comprehensively reviewed in [85]. However, an element of caution must be introduced to this approach because differentiating between adaptive and neutral genes can be problematic in expanding populations: expansions can impact neutral allele frequencies in ways that are similar to the effects of directional selection [86]. In addition, false positives are common even with the most rigorous analytical methods [87].

The ease with which data can now be simultaneously collected for many markers means that studies of discordant genetic differentiation (*i.e.*, the identification of outlier loci) have increased in recent years. A growing number of these studies are based on a genome scanning approach, which means that hundreds or even thousands of markers are used to sample broadly from across the genome (as opposed to a handful of microsatellite loci), and this increases the likelihood of identifying markers linked to genes that are under the influence of natural selection. A number of such studies have been based on dominant markers, specifically amplified fragment length polymorphisms (AFLPs) (e.g., [88–90]). More recently, studies have also been taking advantage of the advent of high-throughput SNP genotyping technologies which can generate data from thousands of markers [91,92] (but see [93] for a discussion of some of the challenges associated with using SNPs).

Adaptive genes can also be inferred from clinal gradients in allele frequencies, which arise when allele frequencies vary along an environmental cline in a seemingly adaptive manner. Studies that have identified such clines sometimes target specific genes that may be expected to show signatures of natural selection. One example of this was the discovery of a latitudinal gradient in Chinook salmon (*Oncorhynchus tshawytscha*) clock gene allele frequencies which corresponded to latitudinal variation in reproductive timing; because clock genes are known to be involved with the regulation of circadian rhythm, the authors of this study had an a priori reason to expect that such a cline may exist [94]. Another approach is to use genome scans to search for genes that may be correlated with environmental clines; as with outlier detections, these scans are increasingly based on high-throughput genotyping of hundreds or thousands of SNP markers. This formed the basis of a study of loblolly pine (*Pinus taeda*) sampled across its range: the frequencies of several SNPs, identified from a total of 1730 loci, corresponded with aspects of geography, temperature, growing degree-days, precipitation and aridity [95]. The authors were then able to assign putative function to a number of SNPs by using annotated orthologs from *Arabidopsis*. Several SNPs that were correlated with climatic variables (such as temperature and precipation) were located within abiotic stress response genes ranging from transmembrane proteins to proteins involved in sugar metabolism.

A note of caution about using clinal patterns to infer patterns of selection is that random events or processes such as founder effects, isolation by distance, or secondary contact of populations that have previously differentiated by genetic drift can create an illusion of an adaptive cline [96]. As with outliers, conclusions may be strengthened by common garden experiments or geographically independent replicates. The latter approach revealed that an insulin signalling gene, the Insulin-like Receptor (InR), had replicate latitudinal clines in allele frequencies among *Drosophila melanogater* populations in both Australia and North America [97]. Replicate findings also strengthened conclusions regarding parallel temperature-associated clines in SNPs which were found in Atlantic cod (*Gadus morhua*) populations in the eastern and western north Atlantic: in both regions, allele frequencies at temperature-associated loci were significantly correlated with the ocean temperature, whereas neutral markers showed no such correlation [98]. See also Box 2 for other approaches that can be used to infer natural selection from genetic data; these are summarized in Table 2.

Box 2 **Genetic signatures of selection**

Even in the absence of broad geographical sampling, evidence for natural selection may be found in patterns of mutation. The rate of evolution in protein-coding genes is commonly assessed using two quantities: d*N* (rate of nonsynonymous substitutions per nonsynonymous site, also called *Ka*) and d*S* (rate of synonymous substitutions per synonymous site, also called *Ks*; [99]). Synonymous substitutions usually occur in the third position of each codon within a gene, and do not alter the encoded amino acid. In contrast, nonsynoymous substitutions (which usually result from a mutation in the first or second position within a codon) alter the encoded amino acid, and are therefore more likely to be deleterious; thus, nonsynonymous substitutions are more likely to be purged from the gene pool via purifying selection. As a result, genes under the influence of purifying selection are expected to have a relatively low number of nonsynomymous substitutions relative to synonymous substitutions (this is referred to as the d*N*:d*S* ratio), while genes not under the influence of selection are expected to have a d*N*:d*S* ratio of approximately 1:1. Conversely, if d*N*:d*S* is greater than 1, positive selection may be acting on the coding region in question (first proposed by [100]; see also [101–103]).

The strategy of d*N*:d*S* comparisons was originally developed to compare sequence evolution between orthologs from different lineages, and polymorphisms within lineages were ignored [104]. For example, Nam, *et al.* [105] estimated rates of non-synonymous and synonymous substitutions in the chicken and zebra finch genomes using one lizard and three mammalian species as outgroups. The authors identified 11,225 orthologs between the two avian genomes. Overall the ratio d*N*:d*S* was 0.152 according to the pairwise comparison between chicken and finch, indicating widespread purifying selection. The authors then sought to identify genes (and their associated functional categories) that were under positive selection in only the chicken or finch genome. Nine hundred and thirty-six genes showed signatures of positive signatures of selection (d*N* > d*S*) in the finch lineage, and 883 in the chicken lineage.

Extended models of nucleotide evolution have been used to investigate possible instances of more recent selection acting within and among populations. Instead of considering only differences between species, these models incorporate data on synonymous and nonsynonymous polymorphism within populations, compared to the number of fixed differences between populations. Under such models, balancing selection and diversifying selection are both expected to maintain an excess of mid-frequency alleles within populations. If balancing selection is acting across populations of interest and their outgroups, a deficit of fixed differences between lineages is expected. Conversely, in the case of diversifying selection, no shared polymorphisms are expected between lineages. For example, Ersoz, *et al.* [106] sequenced 41 candidate genes (thought to be involved in plant-pathogen interactions) from 32 haploid seed megagametophytes of loblolly pine (*Pinus taeda*), using two Scots pine (*Pinus sylvestris*) seed samples as outgroups. The authors proposed various expectations regarding patterns of nucleotide diversity within these candidate gene regions, based on various models of co-evolution between plants and their pathogens. They predicted, for example, that if loblolly pine and their pathogens were engaged in an evolutionary arms race, plant genes involved in resistance would continually develop novel nonsynonymous alleles that would subsequently be fixed, resulting in successive selective sweeps over evolutionary time. Under this scenario, the authors expected to see an excess of nonsynonymous substitutions involved in resistance (directional selection), and a low level of nucleotide diversity (indicative of a selective sweep; the implications of selection on genetic diversity are discussed more extensively in [107]). The authors found that four of the 41 candidate genes examined met the expectations of the arms race hypothesis.

Caution needs to be used in the application of neutrality tests based on d*N*:d*S* estimates for population level inferences because the inferences that can be drawn from the data are not always clear [108,109]. For example, negative selection against slightly deleterious nonsynonymous mutations can lead to a relative excess of rare variants in a population, and this can be confused with balancing or diversifying selection. Also, spurious signals of selection can be detected because demographic processes (for example small population sizes or population bottlenecks followed by expansion) can sometimes lead to the fixation of slightly deleterious alleles as a result of genetic drift.

Signatures of selection may also be inferred by measuring linkage disequilibrium (LD) across the genome. Selective sweeps are expected to be associated with a high degree of linkage disequilibrium around the locus under selection, and (based on the principle of genetic hitchhiking; [110]) long haplotypes are expected to reflect recent selective sweeps; in other words, adaptive alleles are swept rapidly to fixation, and there is insufficient time for recombination to break up surrounding nucleotide combinations (e.g., [111–113]). However, long haplotypes are expected to break up relatively quickly over evolutionary time, so older selective sweeps may not be easy to detect using this approach.

Studies of the human genome provide some of the most widely cited examples of the use of linkage disequilibrium for the identification of regions under selection. For example, Sabeti *et al.* [113] first used this approach to study LD around two loci implicated in human resistance to malaria. The authors compared actual patterns of LD around these loci to expectations that were generated based on simulations that accounted for demographic patterns under neutral models of evolution. They found that haplotypes in the regions of these two loci were much longer than expected according to neutral models.

### 3.2. Model-Based Advances

As noted above, one large stumbling block in the identification of non-neutral markers has been the difficulty in accounting for complex population demography, including historical patterns of population expansion and contraction, unequal migration rates between populations, and inbreeding. A failure to account for such demographic processes can lead to either spurious signatures of selection at loci that are in fact neutral [86], or to a lack of power to detect loci under selection. Advances in the sophistication of statistical tools and models available for the analysis of molecular data are facilitating a much more intricate and comprehensive understanding of the processes that shape neutral and adaptive genetic variation. For example, currently available Bayesian approaches [114,115] and coalescence models (e.g., [116,117]) incorporate relatively realistic scenarios in which the migration rate can differ between pairs of subpopulations, and multiple historical population bottlenecks and expansions can be accounted for. Additionally, a number of software packages incorporate spatial and environmental data with genetic data to identify loci that are associated with specific environmental variables (e.g., [118]). Many of these model-based advances are reviewed in detail by [119].

### 3.3. Isolation by Adaptation

Finally, when examining patterns of population differentiation at neutral *versus* non-neutral loci, it is important to keep in mind that gene flow will not necessarily ensure that non-adaptive genes are continually exchanged between even proximate populations. Although the differentiation of neutral markers is driven primarily by stochastic processes, whereas that of non-netural markers is driven by both selective and stochastic processes (e.g., [120]), natural selection can also influence the distribution of markers that are neither being directly selected, nor are linked to regions under selection [121]. This arises if divergent selection is sufficiently strong to promote reproductive isolation between populations. In these cases, a reproductive barrier will then create a barrier to gene flow that results in the potentially genome-wide differentiation of populations following genetic drift. This will lead to an inverse correlation between gene flow and the adaptive divergence of populations, and thus a positive association between the phenotypic divergence and neutral molecular genetic differentiation of populations following a pattern that is known as isolation by adaptation (IBA) [122,123]. In other words, populations will not only diverge at adaptive loci as a direct result of selection, but will also diverge at neutral loci as a direct result of drift, which is indirectly a result of selection via a reproductive barrier. This pattern was identified in a semi-natural experiment in which adjacent populations of sweet vernal grass (*Anthoxanthum odoratum*) had diverged from one another as a result of adaptation to different nutrient additions. Genetic differentiation was evident at outlier loci, and also across a wider survey of putatively neutral loci. This was interpreted as evidence that the selection pressures from varying combinations of nutrient additions in different plots was strong enough to cause reproductive isolation between populations, which in turn has led to neutral genetic differentiation as a result of genetic drift [89]. Studies such as this, which have identified candidate adaptive molecular markers, should in the future find it increasingly feasible to take the next step and characterize the phenotypic outcomes of alternative genotypes (Box 3).

Box 3 **Linking genotype to phenotype**

The identification of outlier loci, or candidate genes under selection, must always retain an element of speculation until the genetic region in question has been directly linked to a phenotype that is subject to selection. Both quantitative trait locus (QTL) mapping and genome-wide association studies are used to identify correlations between specific marker alleles and phenotypic traits of interest. QTL mapping is perhaps the oldest form of genome scanning, and has been widely used in studies of genetic model organisms and commercially important species for at least two decades (e.g., [124]). The aim of QTL analysis is to use a large number of individuals from a known pedigree that show considerable variation in phenotypic trait(s) of interest, and to genotype them across a large number of loci using a set of markers that cover the whole genome. Usually QTL mapping is carried out using an F_2_ or backcrossed family (BC) from a known cross, or sometimes using recombinant inbred lines (RILs). A linkage map is constructed based on observed rates of recombination between markers in the mapping population, and measurements of phenotypic traits are made from the mapping population under standardized conditions. Various statistical methods are used to calculate estimated recombination rates between marker loci and the QTL that control for the phenotypic trait(s) of interest. Depending on the genetic architecture of the trait and the experimental design, one or more QTL can be identified and the relative proportion of phenotypic variation explained by each QTL can be calculated. Also, interactions between different QTL can sometimes be identified (e.g., epistasis or pleiotropy). For example, Latta *et al.* [125] developed 179 RILS from a cross between moist- and dry-associated ecotypes of *Avena barbata* (wild oats). Two loci accounted for more than half of the variation in plant fitness across both moist and dry environments, and no genotype-by-environment interactions were detected with regard to the direction of selection at these loci.

Studies that link phenotypes to genotypes have recently been extended on a more widespread scale to populations of unrelated individuals, in the form of genome-wide association (GWA) studies. GWA studies have been widely used in the study of human disease (recently reviewed by [126]), but have only more recently been applied to other organisms (e.g., *Arabidopsis*, [127,128]; dogs, [129]). GWA studies generally provide higher resolution than traditional QTL studies, because recombination between loci is generally greater in large populations of unrelated individuals than that in F_2_ or BC families. A recent GWA study of 15 different morphological traits in barley incorporated 500 different cultivars that were genotyped with 1536 SNPs [130]. The authors identified 18 genomic regions associated with the 15 traits (most of the traits were associated with a single genetic locus). Based on these results, the authors selected one phenotypic trait–anthocyanin pigmentation, which is involved in determining seed colour—for more detailed fine-scale mapping using a QTL approach after crossing two of the cultivars included in the original GWA study to create a mapping population. This allowed them to identify the specific mutation involved in generating the variation explained by the candidate locus identified in their original GWA study.

### 3.4. Future Work

There are a number of exciting avenues for future research that will allow researchers to increasingly incorporate data on adaptive genes into studies of molecular ecology. The sequencing of a greater number of genomes from non-model organisms will be among the most obvious and rapid advancement in genomics over the coming years, and this will provide opportunities for the identification of non-neutral markers in numerous and diverse species. Furthermore, cross-validation using a combination of different approaches will lead to a greater understanding of the interaction between demographic processes and selection, the interaction between selection at linked loci in the genome, and fine-scale patterns of molecular evolution. Studies of signatures of selection in the human genome have led the way in this regard (reviewed by [131]), and can serve as models for similar studies in other organisms.

There are two widespread challenges that arise in many studies that target genes that are under the influence of selection. First, studies in non-model organisms now frequently hone in on relatively broad genomic regions that are under selection, but it remains difficult to actually identify the genes (or the mutations) that are subject to selection. Increasing the density of markers in genome scans is paramount to overcoming this problem, and validating signals of selection from particular genes using multiple methods should also help. Second, once a candidate gene has been identified, it may have no known annotated function. This occurs because annotated functional genes from model organisms may not overlap with genes that are under the influence of selection in non-model organisms that are being studied in the context of ecological and evolutionary genomics. Advances in identifying the functional significance of genes subject to selection will require ongoing integration between genomics methods and functional experiments that provide mechanistic insights into molecular pathways controlled by candidate genes (reviewed by [132]). Studies of gene expression, which can be carried out using microarrays, quantitative PCR (qPCR), and comparative sequencing of the transcriptome, can also provide evidence of differential expression of candidate genes. Genome wide scans based on complete genomic data—already close to fruition in humans—will permit a much more detailed understanding of fine scale processes involved in genome evolution (reviewed by [131]). Finally, future work on adaptive genes will likely also focus on epigenetic modifications in DNA methylation and DNA-associated proteins such as histones, which can vary among individuals and populations of the same species. The heritability of some of these modifications is now widely accepted, and means that heritable variation in ecologically important phenotypic traits may be apparent even in the absence of DNA polymorphisms (see [63]). We are therefore entering a truly exciting time in molecular ecology, in which we seem poised to make numerous important discoveries about the interactions between genotypes and phenotypes in varied—and often rapidly changing—environmental conditions.

## 4. Conclusions

To date, the vast majority of studies in the field of molecular ecology have been based on neutral molecular markers, in other words genetic regions that do not directly influence fitness. These markers have given us invaluable insights into parameters such as genetic diversity within populations, genetic differentiation among populations, inbreeding, and demographic events; however, they provide limited insight into adaptive evolution and evolutionary potential. In recent years, developments such as next-generation sequencing mean that we have become increasingly able to develop non-neutral markers by targeting genetic regions that are directly influenced by natural selection, which means that a growing number of studies have been able to use molecular genetic data to directly study natural selection and local adaptation of natural populations from a wide range of taxonomic groups. In addition, researchers are increasingly able to link genotypes to phenotypes under a range of environmental conditions. More specifically, these data have provided numerous examples of how local adaptation shapes the genetic diversity and differentiation of populations, and have also provided insight into some of the mechanistic processes behind inbreeding depression, and some of the demographic processes that are associated with adaptive evolutionary change. Although researchers will continue to use neutral molecular markers because of their ease of use and their relatively straightforward histories (which can allow more accurate inferences of past demographic events), future studies will be increasingly likely to supplement data from neutral markers with data from markers that are influenced by natural selection.

## Figures and Tables

**Table 1:** Some of the candidate genes and the phenotypic traits that they influence in natural populations of non-model species. Adapted from [23].

Candidate Gene	Species	Phenotypic Trait	Reference
Calmodulin (CALM1)	Darwin’s finches (*Geospiza* spp.)	Beak morphology	[24]
cGMP-dependent protein kinase (KGP1)	Honey bee (*Apis mellifera*)	Foraging behavior division of labour	[25]
Pantophysin (PAN1)	Cod (*Gadus morhua*)	Growth	[26]
Phosphoglucose isomerase (PGI)	Glanville fritillary butterfly (*Melitaea cinxia*)	Dispersal	[27]
Protein tyrosine phosphotase (PTEN)	Nasonia wasps	Longevity/incompatability	[28]
Thyroid hormome receptor alpha	Ambystomatid salamanders	Timing of metamorphosis	[29]
Wingless	Heliconius butterflies	Wing patterning	[30]
Tyrosine related protein kinase I (TRYP1)	Soay sheep (*Ovis aries*)	Coat colour polymorphism	[31]
Gp-9-odorant binding protein precursor	Fire ants	Social organization behaviour	[32]

**Table 2:** A summary of approaches used to identify genomic regions under the influence of selection.

Approach	Target Region	Strategy
Identify regions under selection based on genomic information only	Protein-coding regions	Compare rate of nonsynonymous (d*N*) *versus* synonymous (d*S*) substitutions (neutrality test) between species or populations
	Whole Genome	Linkage-disequilibrium based approaches Population Differentiation Levels Comparisons of Nucleotide Diversity between different genome regions (not discussed in detail here)

Search for correlations between phenotypes and allele frequencies	Whole Genome	QTL mapping Genome-Wide Marker Association (GWA) Studies
Search for correlations between environmental variables and allele frequencies.	Whole Genome	Genome Scanning combined with the identification of outlier loci.
